# Quantification of Circulating Cell-Free DNA as a NETosis Marker in Trauma Patients with Type 2 Diabetes Mellitus

**DOI:** 10.3390/mps8020042

**Published:** 2025-04-16

**Authors:** Filiz Sahin, Regina Breinbauer, Caren Linnemann, Melike Tombaz, Andreas K. Nussler, Sabrina Ehnert

**Affiliations:** Siegfried Weller Institute for Trauma Research, BG Unfallklinik Tübingen, Eberhard Karls Universität Tuebingen, 72076 Tuebingen, Germany; filiz.sahin.7@gmail.com (F.S.); regina-breinbauer@gmx.de (R.B.); andreas.nuessler@gmail.com (A.K.N.)

**Keywords:** neutrophil extracellular traps, trauma, diabetes mellitus, NET marker

## Abstract

Type 2 diabetes mellitus (T2DM) significantly impairs fracture healing, with neutrophils playing a crucial role in this process. In T2DM, these immune cells are over-activated, leading to the excessive release of neutrophil extracellular traps (NETs), increasing inflammation and hindering recovery. Thus, a need for markers to assess patients in the risk group arises. This study demonstrates that circulating cell-free DNA (cfDNA) can be efficiently quantified from serum samples by a single-step qPCR and be used as a marker for NETosis. Our results revealed that trauma patients with T2DM have the highest cfDNA levels, followed by trauma patients, and the healthy group has the lowest. The method shows strong correlations between cfDNA and neutrophil-specific markers such as MPO, citH3, AZU1, and α-defensin, highlighting its potential as a rapid indicator of NETosis. This approach could allow the timely interference for high-risk patients, ultimately improving healing outcomes and reducing complications such as chronic inflammation, non-union fractures, and diabetic foot ulcers.

## 1. Introduction

Neutrophils are the most abundant innate immune cell type and first responders; therefore, neutrophil defense mechanisms are crucial to fighting inflammation. One of these mechanisms is the release of neutrophil extracellular traps (NETs). NETs are structures released from neutrophils by NETosis, which has two fundamental mechanisms. In “suicidal” NETosis, cells are programmed to die while releasing the NETs. This process might be initiated by various cell signals, such as reactive oxygen species (ROS) generation and Ca^2+^ influx [1]. During suicidal NETosis, which is dependent on NADPH oxidase 2 (NOX2), protein–arginine deiminase type-4 (PAD4) catalyzes the citrullination of histones, followed by chromatin decondensation. DNA is released from the cell comprising antimicrobial proteins, such as neutrophil elastase (ELA2/NE), myeloperoxidase (MPO), and citrullinated histone H3 (citH3). ELA2 and MPO also work together to drive chromatin decondensation in addition to their antimicrobial properties. The second mechanism, called “vital” NETosis, is a quick response and is also dependent on the activation of PAD4 through Ca^2+^, which in turn leads to histone citrullination and NET release by vesicles. Even though the mentioned types are mostly accepted as the modes of action, NETosis is a complex response with many triggers and mechanisms, such as NOX-independent NETosis or various other histone modifications [1,2,3].

Despite the fact that NETs are innately anti-inflammatory, in some cases, they can become over-activated and turn into a risk factor [4]. One of these instances is diabetes mellitus (DM) [5,6]. DM is a metabolic disease characterized by hyperglycemia and has been linked to delayed fracture and wound healing [7]. A comprehensive study focused on disability-adjusted life years (DALYs) showed that in 29 years, DM has risen from the 20th to the 8th most common cause of DALYs, representing the loss of one year of full health [8,9]. A study performed in BG Unfallklinik Tübingen, Germany, showed that 12.4% of all the inpatients were diagnosed with DM [10], with a rising prevalence since the mentioned study was conducted. Patients with DM had more comorbidities, such as coronary heart disease, chronic kidney disease, and hypertension. In addition, patients with DM had significantly extended hospital stays, which results in an economic burden to the patients and the health system [11]. Worldwide, the prevalence of diabetes has been increasing, and the number is expected to rise to 700 million adults (20–79 years of age) by 2045 if the same trend of a sedentary lifestyle and unhealthy diet persists [12]. When undiagnosed diabetic and pre-diabetic individuals are taken into consideration, the actual number of patients experiencing complications is expected to be substantially higher. It has been reported that patients with T2DM have a higher expression of PAD4, and higher basal levels of NETs compared with healthy individuals [13]. Even under glycemic control, patients with T2DM showed elevated levels of MPO–DNA complexes, which strongly indicates NETosis [14]. Another study demonstrated that patients with persistent diabetic foot ulcers have increased NET components circulating in the blood, which was correlated with delayed wound healing [15]. In the light of these findings, focusing on patients with type 2 DM (T2DM) and their risk factors, such as NETosis, is crucial.

NET detection and quantification can be performed using numerous methods. These methods usually depend on NET-specific markers such as MPO, citH3, ELA2, or various cytokines rather than directly detecting the released DNA [16]. Furthermore, methods like ELISA or Western Blot are time-consuming, whereas quicker methods like flow cytometry are costly [1]. A method is needed for the rapid and resource-efficient screening of NET release, particularly in clinical settings, to assess patients at high risk for chronic morbidities. This method should not require extensive sample preparation or a complex protocol. In this manuscript, we describe a streamlined quantitative PCR (qPCR) method without prior DNA isolation to quantify the circulating cell-free DNA (cfDNA) in patient serum samples as a marker for NETosis.

## 2. Materials and Methods

### 2.1. Study Population

Venous blood was collected from volunteers after obtaining consent in accordance with the Declaration of Helsinki. This study was approved by the Ethics Committee of the Medical Faculty of the University Hospital and the Eberhard Karls University of Tübingen on 9 July 2020, with approval number 346/2015-BO2, amended on 30 March 2020. Blood samples were collected from 67 trauma patients, 31 trauma patients with DM, and 27 control subjects. All patients were admitted to the Septic Trauma Surgery Department, BG Clinic, Tübingen. Control subjects were either healthy volunteers or admitted patients without trauma, inflammation, or DM. Information about the cohort, including relevant blood markers, is presented in Table 1.

### 2.2. Sample Collection

For the venous blood collection, serum collection tubes (S-monovette 7.5 mL Z, Sarstedt, Nümbrecht, Germany) and plasma tubes with EDTA, Li-heparin, and citrate (S-monovette, Sarstedt, Nümbrecht, Germany) were used. After taking the blood, the tubes were placed upright for 30 min at room temperature, then centrifuged at 1000× *g* for 10 min at room temperature. Serum and plasma layers were collected and stored at −80 °C until further use.

### 2.3. Neutrophil and NET Isolation

Neutrophils were isolated from freshly collected venous blood using EDTA tubes (S-monovette 7.5 mL Z, Sarstedt, Nümbrecht, Germany). In total, 6 mL of blood were layered on top of a 6 mL Lympholyte poly separation medium (Cedarlane, Burlington, ON, Canada), then centrifuged at 500× *g* for 40 min at room temperature. The PMN layer was collected into a new 15 mL tube. Cells were washed twice with PBS and centrifuged at 400× *g* for 10 min at room temperature. The acquired neutrophil pellet was resuspended in an RPMI medium (Sigma-Aldrich, Steinheim, Germany), and the cells were counted using a Neubauer counting chamber. NETs were isolated as previously described [17]. Briefly, neutrophils were diluted to 5 × 10^6^/mL and stimulated with 500 nM PMA. Cells were incubated for 4 h at 37 °C and 5% CO_2_. Afterward, the cells were scratched with a cell scraper. The obtained cell suspension was centrifuged at 600× *g* for 10 min at 4 °C. The pellet was discarded, and the supernatant was transferred to another tube and centrifuged at 18,000× *g* for 15 min at 4 °C. The supernatant was removed, and the pellet was resuspended in 10 µL of cold PBS. The DNA content was measured in the FLUOstar Omega plate reader (Software version 5.70, BMG Labtech, Ortenberg, Germany). Isolated NETs were stored at −80 °C and used immediately after thawing.

### 2.4. DNA Isolation from Neutrophils

Isolated neutrophils were centrifuged at 600× *g*, for 10 min at room temperature. After resuspending in 250 µL of 50 mM NaOH solution, the samples were incubated at 98 °C at 250 rpm in the thermoshaker for 30 min. Then, the samples were frozen at −80 °C and thawed again. Next, 25 µL of 1 M TRIS solution at pH = 8 and 250 µL of ddH_2_O were added to the samples and centrifuged at 14,000× *g* at 4 °C for 10 min. The supernatant was transferred to a new tube and stored at −20 °C.

### 2.5. Absolute Quantification of NETs by the Standard Curve Method

NETs were diluted in a 1:2 ratio in PBS to create a standard to be used in the absolute quantification of cfDNA from the serum samples. The starting DNA concentration of the pooled isolated NETs from three donors was 39.22 ng/mL, ending the dilution at a 2.42 ng/mL concentration, as measured spectrophotometrically using the FLUOstar Omega plate reader (Software version 5.70, BMG Labtech, Ortenberg, Germany). qPCR was performed with 10 µL of GreenMasterMix (2X) High ROX (Genaxxon Bioscience, Ulm, Germany), a working concentration of 1 pmol/µL from the forward and reverse KRAS primer (Table 2), 4 µL of nucleic acid-free water, and 2 µL of isolated NETs per well, in triplicates. The initial denaturation step was at 95 °C for 1 min, then 40 cycles of 95 °C for 15 s, followed by 60 °C for 30 s, and 72 °C for 30 s. The results were used to prepare a dilution standard for the quantification of cfDNA in serum samples.

Next, the circulating cfDNA in the serum samples was quantified using qPCR for the KRAS gene [18] (Table 2) as the NETosis indicator. A total of 20 µL of qPCR mix were composed of 10 µL GreenMasterMix (2X) High ROX (Genaxxon Bioscience, Ulm, Germany), 2 µL of 1 pmol/µL of each primer, and 2 µL of nucleic acid-free water per well. In total, 4 µL of serum/plasma samples were added directly to the reaction mix in the wells. Thermal cycling started with initial denaturation at 95 °C for 1 min, followed by 40 cycles of 95 °C for 15 s, 60 °C for 30 s, and 72 °C for 30 s. The reaction specificities were checked by melting curves. Every sample was analyzed in triplicate. The concentrations were then calculated using the linear regression of the standard curve [19].

### 2.6. DNA Isolation from Serum Samples

DNA was isolated from serum samples using the All-In-One DNA/RNA Mini-Preps Kit (Bio Basic, Markham, ON, Canada). A total of 500 µL of serum samples were processed per the manufacturer’s instructions. The DNA concentration was assessed using the FLUOstar Omega plate reader (Software version 5.70, BMG Labtech, Ortenberg, Germany).

### 2.7. Fluorometric Quantification of DNA in Serum Samples

The DNA content of the serum samples was quantified using fluorescent staining. An amount of 1 µM of Sytox Green (Thermo Fisher, Waltham, MA, USA) was added to the serum samples and incubated for 5 min at room temperature and in the dark. The fluorescence was measured using the ClarioStar plate reader (Software version 5.70 R3, BMG Labtech, Ortenberg, Germany). For the experiments with the DNA addition to the serums to check the sensitivity of the fluorometric quantification, the isolated DNA sample was serially diluted in a 1:2 ratio, the concentration starting from 135.60 and ending at 0.52 ng/mL. The DNA concentration was quantified with the plate reader. The reading from the serum sample without DNA addition was used to normalize the effect of the cfDNA that was already found in the serum. Moreover, according to the qPCR experiments, the serum samples with the least cfDNA concentrations were chosen to minimize this effect.

### 2.8. ELISA

The protein concentrations were measured using ELISA kits per the manufacturer’s instructions. The measurements were taken using the FLUOstar Omega plate reader (Software version 5.70, BMG Labtech, Ortenberg, Germany). The optical density was determined at a 450 nm wavelength and corrected with 540 nm. Information about each kit is given in Table 3.

### 2.9. Statistical Analysis

The data were analyzed using the non-parametric Kruskal–Wallis test or two-way ANOVA with Tukey’s multiple comparison test. Box plots are generated using Tukey’s modification. The analysis was conducted using GraphPad Prism Version 8 (La Jolla, CA, USA). *p* < 0.05 was considered statistically significant. “N” indicates the biological replicates, while “n” indicates the technical replicates. The Spearman correlation analysis was conducted using the psych package in R to evaluate the relationships among the biomarkers. The correlation matrices and significance levels were extracted, and correlation plots were generated with the corrplot package in R. Detailed information about the statistical tests is indicated in the figure legends. K-means clustering was applied to the biomarker data to investigate the dataset’s structure and identify potential subgroups. The results were visualized through a principal component analysis (PCA) using the factoextra package, with the first two principal components capturing the majority of the variance. The cluster assignments were further illustrated using ggpubr and ggscatter, with the patient groups represented with distinct shapes and clusters by unique colors. The correlation and principal component analyses were performed in R (version 4.4.0).

## 3. Results

### 3.1. Study Cohort Characteristics

**Figure 1:**
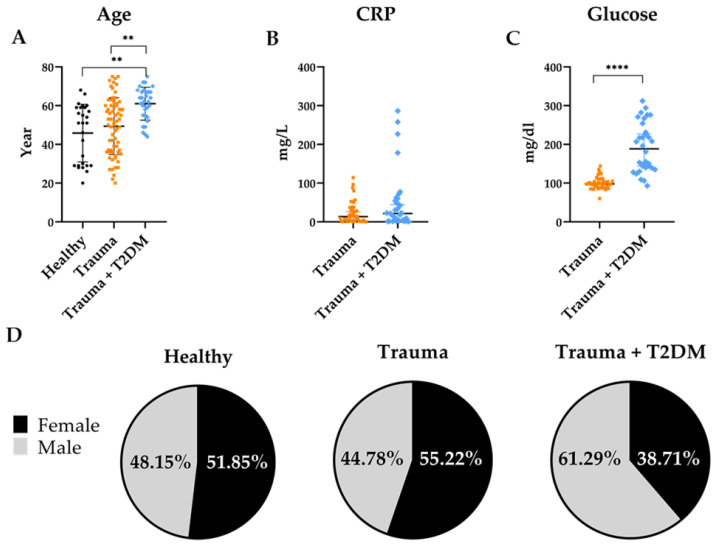
Cohort characteristics. (**A**) Age distribution of all participants, (**B**) CRP levels, and (**C**) blood glucose levels of the patients. (**D**) Gender distribution of the cohort. Healthy N = 27, Trauma N = 67, Trauma + T2DM N = 31. Statistical analysis was conducted using the Kruskal–Wallis test (**A**), and the Mann–Whitney test (**B**,**C**). ** *p* < 0.01; **** *p* < 0.0001.

The study involved three groups: healthy volunteers, trauma patients without T2DM, and trauma patients with T2DM. Patients with trauma and T2DM were significantly older than both trauma patients without T2DM and healthy volunteers. However, no significant age difference was found between the healthy volunteers and trauma patients without T2DM (Figure 1A). This suggests that T2DM might be associated with older age in patients with trauma, which is not surprising given that T2DM prevalence increases with age.

C-reactive protein (CRP) levels did not show any significant differences between the trauma and trauma + T2DM groups (Figure 1B), eliminating the possibility of differently increased cfDNA amounts related to inflammation between the two groups. Random blood glucose levels were significantly elevated in the trauma + T2DM group compared with the trauma group without T2DM (Figure 1C).

Regarding gender distribution, the healthy volunteer group comprised 51.85% females and 48.15% males. Within the trauma group, 55.22% were female and 44.78% were male, while in the trauma + T2DM group, females represented 38.71% of the participants (Figure 1D). The gender distribution ratio between the groups was not significantly different (chi-square df = 2.341; *p* value = 0.3102).

### 3.2. cfDNA Can Be Quantified from Serum Samples Directly

**Figure 2:**
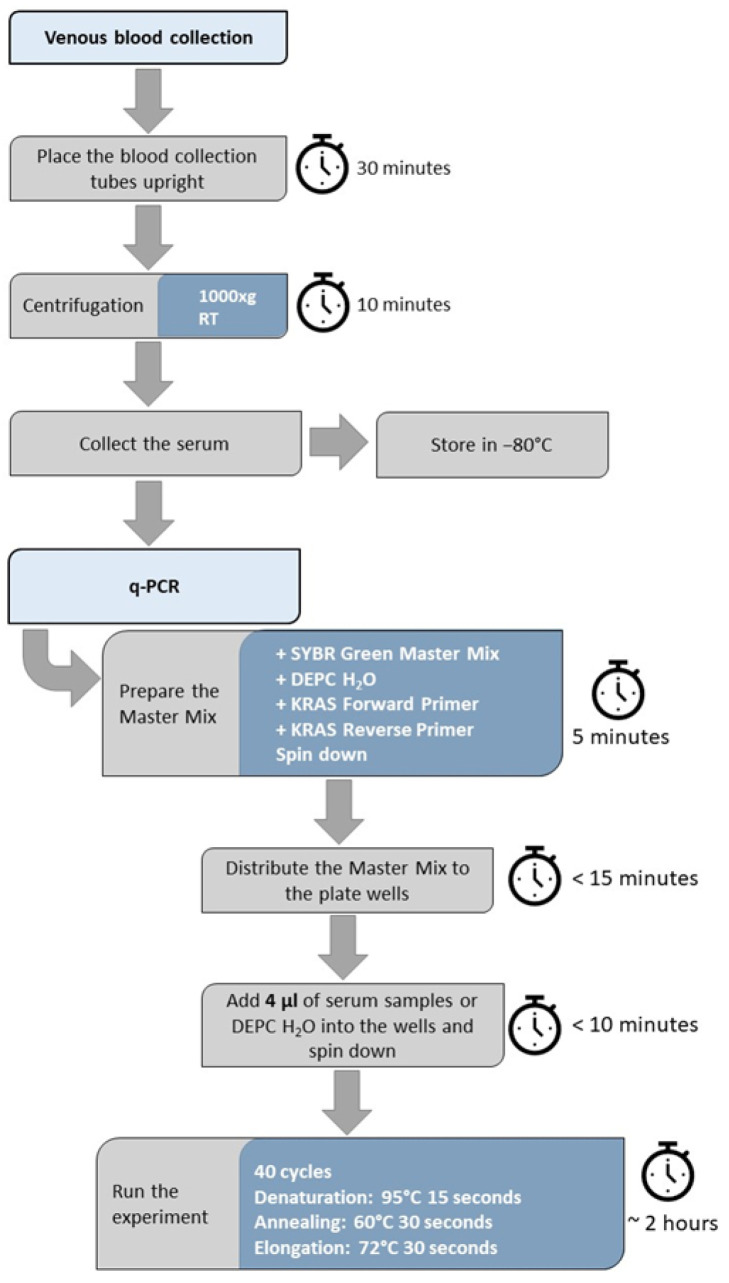
Workflow of the qPCR method, including the processing time for each step.

**Figure 3:**
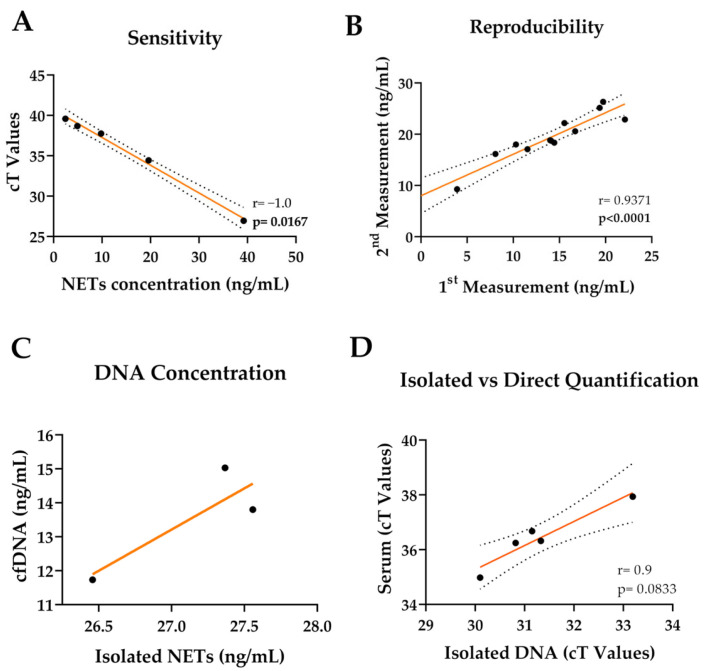
Quantification of cell-free DNA in serum samples. (**A**) A standard for qPCR was prepared to assess the sensitivity of the method and further quantification of the samples. (**B**) The same samples were used two months apart for q-PCR. N = 12, n = 3. (**C**) Correlation between the isolated NETs and cfDNA without isolation from the same donors, measured using qPCR. N = 3, n = 3. (**D**) Correlation of quantification of isolated DNA from serums and directly used serum samples. N = 5, n = 2. Correlation analysis was performed using pairwise Spearman correlation (r). Data points represent the mean of the technical replicates. The dotted lines indicate a 95% confidence interval.

Collected serum and plasma samples were used to quantify the released NET amount as measured by DNA using qPCR without prior isolation (Figure 2). The plasma samples did not give readings from the qPCR experiments; therefore, only the serum samples were used in our further qPCR-based experiments. A standard curve for absolute quantification was composed using known concentrations of isolated NETs, and the detection limit was 2.4–39.2 ng/mL (Figure 3A). To check the sensitivity and stability of the method, the same qPCR experiment was repeated after 2 months with the same samples. During this period, the samples were stored at −80 °C. To rule out the possibility of handling differences, the repetition experiment was conducted by a different researcher. The relationship between the two results showed a robust correlation (Figure 3B), indicating that the storage of the samples or handling by different individuals does not affect the outcome (r = 0.9371). The qPCR method was performed using isolated NETs and serum samples without isolation from the same donors to assess the yield with and without an isolation step. The results show a trend of correlation (Figure 3C). Furthermore, the correlation between the isolated circulating cfDNA samples from the serums and the direct use of serum samples was investigated. The results revealed a strong positive correlation between the isolated cfDNA and the directly used serum samples.

### 3.3. ELISA or Fluorometric Quantification Is Not Sensitive in Low Concentrations

**Figure 4:**
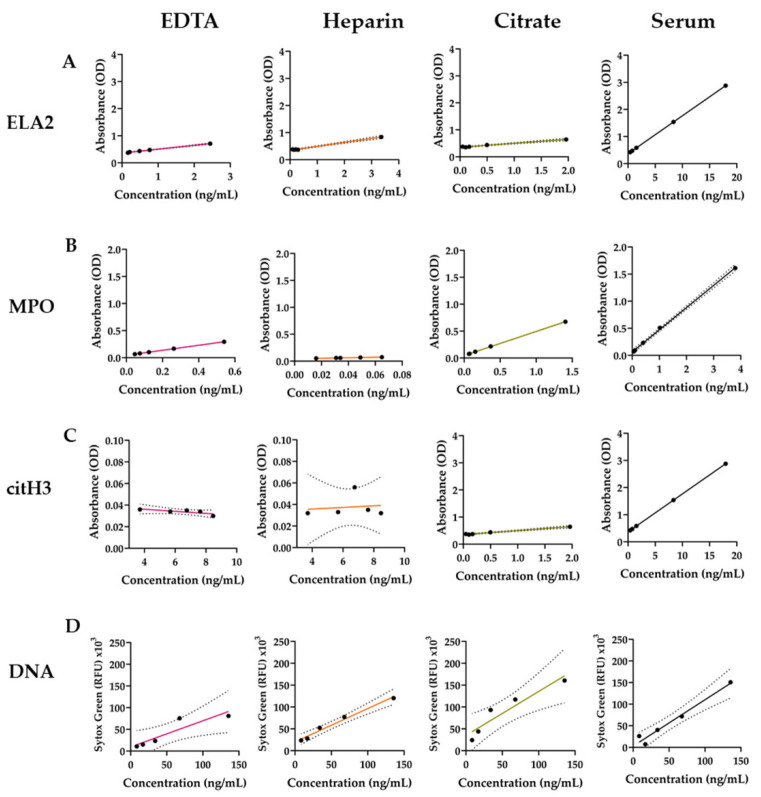
Assessment of the sensitivity of different NET quantification methods. (**A**–**C**) Protein concentrations of the samples collected with different blood collection tubes were measured using ELISA. For each sample group, N = 1, n = 2. (**D**) Fluorometric quantification of DNA in the serum and plasma samples. 1:2 serially diluted DNA samples were added to the serum and plasma samples, stained with Sytox Green, and then quantified with a plate reader. N = 2, n = 2. Data points indicate the mean of the technical replicates. Lines are obtained through the linear fitting of the absorbance values. The dotted lines indicate 95% confidence intervals.

After determining the sensitivity of the qPCR method, other frequently used methods were evaluated. ELISA is a commonly used approach for the quantification of NETosis. The blood samples collected using different types of blood collection tubes were compared with the ELISA analysis. Plasma samples were collected using EDTA, heparin, and citrate blood tubes. Serum samples were collected using serum blood tubes. The proteins associated with NETosis, such as ELA, MPO, and citH3, were chosen for the assessment using ELISA. According to the results, serum samples have the highest sensitivity in ELA2, MPO, and citH3 detections (Figure 4A–C, Table 4). The other three sample-type curves show a difference between all three proteins; however, they have a lower sensitivity for all the kits. The limit of detection (LoD) and the limit of quantification (LoQ) were the lowest with serum samples, except for the MPO kit. The citH3 ELISA kit lost its sensitivity when the sample had a lower concentration.

Next, Sytox Green staining was employed as a common detection method for cfDNA and NETosis [1,20,21,22]. To check the sensitivity of this method with serum and plasma samples, the isolated DNA samples with known concentrations were serially diluted and added to the serum and plasma. The samples spiked with DNA were stained with Sytox Green dye and fluorometrically quantified. The EDTA plasma sample was the least sensitive, showing 60% sensitivity. The most stable plasma sample was heparin, with the lowest LoD and LoQ for the fluorometric method (Figure 4D, Table 4). Overall, the qPCR method had the lowest LoD and LoQ with a high sensitivity (Table 4).

**Table 4:** Limits of detection (LoDs), limits of quantitation (LoQs), and sensitivities of the tested methods. LoD = 3.3 (standard deviation of the response (Sy)/Slope (S)). LoQ = 10 (Sy/S). Sensitivity was calculated using the slopes (y = mx + b) of the linear fitting of the respective methods (Figure 4) and shown as a percentage (m = 1 accepted as 100%) [23].

		ELA2 (ELISA)	MPO (ELISA)	citH3 (ELISA)	cfDNA (Fluorometric)	cfDNA (qPCR)
LoD (ng/mL)	EDTA	0.197	0.005	N/A	90.807	N/A
	Heparin	0.378	0.002	52.356	26.529	N/A
	Citrate	0.334	0.017	8.777	69.217	N/A
	Serum	0.001	0.16	0.972	36.013	0.84
LoQ (ng/mL)	EDTA	0.598	0.015	N/A	275.174	N/A
	Heparin	1.145	0.006	158.655	80.393	N/A
	Citrate	1.012	0.053	26.599	209.751	N/A
	Serum	0.004	0.489	2.947	109.133	2.56
Sensitivity	EDTA	14.3%	46.2%	N/A	60%	N/A
	Heparin	14.5%	46.8%	7.4%	76%	N/A
	Citrate	14.8%	44.6%	8.9%	105%	N/A
	Serum	15.9%	47.9%	16%	107%	98%

### 3.4. cfDNA and NET-Related Protein Concentrations in Trauma Patients

**Figure 5:**
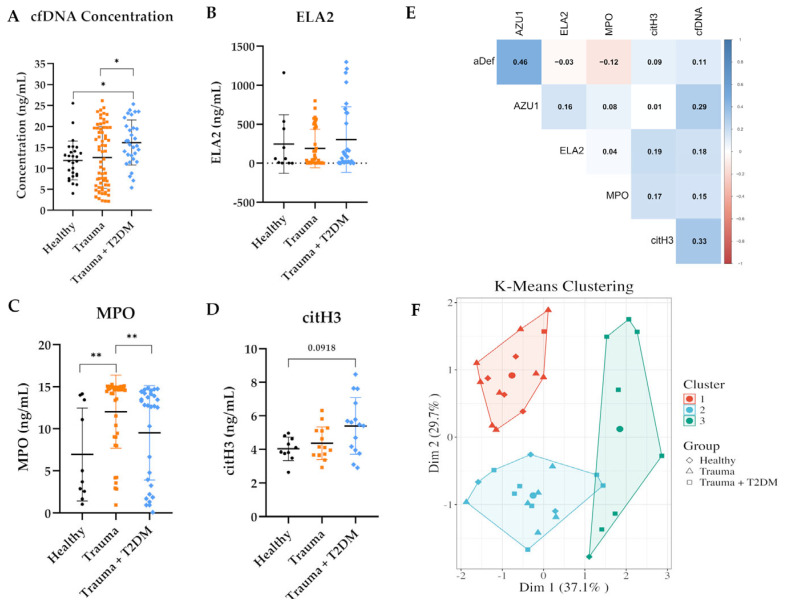
Assessment of the cfDNA concentration and the NETosis-related proteins in the study cohort. (**A**) The concentration of cfDNA in serum samples was determined using qPCR for the KRAS gene. Healthy N = 27, n = 3; Trauma N = 67, n = 3; Trauma + T2DM N = 31, n = 3. (**B**–**D**) Protein concentrations were measured from serum samples with respective ELISA kits. ELA2: Healthy N = 10, n = 2; Trauma N = 40, n = 2; Trauma + T2DM N = 30, n = 2. MPO: Healthy N = 10, n = 2; Trauma N = 40, n = 2; Trauma + T2DM N = 30, n = 2. citH3: Healthy N = 10, n = 2; Trauma N = 15, n = 2; Trauma + T2DM N = 15, n = 2. Data points demonstrate the mean of the technical replicates. Statistical analysis was performed using the Kruskal–Wallis test. * *p* < 0.05; ** *p* < 0.01 as indicated. (**E**) Spearman’s correlation matrix of variables. Correlation coefficients were calculated using the Spearman method in R for complete observations. Blue represents a positive correlation, whereas red represents a negative correlation. (**F**) K-means results via PCA components. In the resulting plot, patient observations are represented by shapes, and using principal components, concentration ellipses around each cluster are drawn. The circles in the middle represent centroids. Diamonds represent healthy controls N = 7, triangles represent trauma patients N = 15, and squares represent trauma patients with T2DM N = 14.

The qPCR method was utilized with trauma patients. The concentration of cfDNA in each serum sample was calculated using the standard curve (Figure 3A). The group of patients with trauma and T2DM had the highest amount of cfDNA in their serum, followed by trauma patients and healthy controls with the least amount of cfDNA (Figure 5A). The average cfDNA concentration was 11.8 ng/mL for the healthy group, and 12.5 ng/mL and 16.15 ng/mL for the trauma and trauma + T2DM patients, respectively. Next, the NETosis-related proteins were quantified using ELISA to evaluate if the quantified cfDNA reflects the amount of NETosis. ELA2, an antimicrobial protease [24], had similar levels in all three sample groups (Figure 5B). Another antimicrobial neutrophil protein [24], MPO, showed a significantly higher concentration in trauma patients than in healthy controls (Figure 5C). citH3 facilitates chromatin condensation during NET release [25] and is frequently used as a NETosis marker. Here, the highest concentration of citH3 in serum was in the trauma + T2DM patient group, followed by only the trauma and healthy groups (Figure 5D). This clear trend in citH3 serum concentration is in line with cfDNA concentrations. To rule out the possibility that the observed increase in cfDNA is due to patient age, a correlation analysis was performed between cfDNA concentrations and age. The analysis revealed no correlation between age and measured cfDNA (Supplementary Figure S1A). Moreover, an age-matched sub-cohort was analyzed to eliminate age’s potential influence on NETosis. The trends in the cfDNA and protein concentrations remained unchanged, if not clearer, showing the highest concentrations in trauma with the T2DM patient group (Supplementary Figure S1B–D).

To check if the measured cfDNA amounts are associated with NET release, the correlation coefficients for the NET marker proteins and cfDNA concentrations were calculated. Neutrophil proteins MPO, ELA2, citH3, AZU1, and α-defensin were selected for this correlation. The cfDNA concentration showed a positive correlation with all the neutrophil-related proteins. Importantly, this correlation was significant with α-defensin and AZU1 (Figure 5E). This correlation with the distinguished experimental groups also demonstrated a positive correlation with multiple NETosis markers, albeit the small cohort number (Supplementary Figure S2). Then, the cfDNA, MPO, citH3, and ELA2 concentrations were used for the k-nearest neighbors clustering for the subjects. From this clustering, three groups emerged. Cluster 1 contains a mix of healthy individuals and trauma patients, whereas Cluster 2 overlaps all three groups, indicating heterogeneity. However, Cluster 3 primarily consists of trauma + T2DM patients, suggesting distinct differences in the NETs marker proteins, which might be influenced by the presence of diabetes in patients with trauma (Figure 5F).

### 3.5. Correlation of qPCR and Fluorometric Quantification in Serum Samples

**Figure 6:**
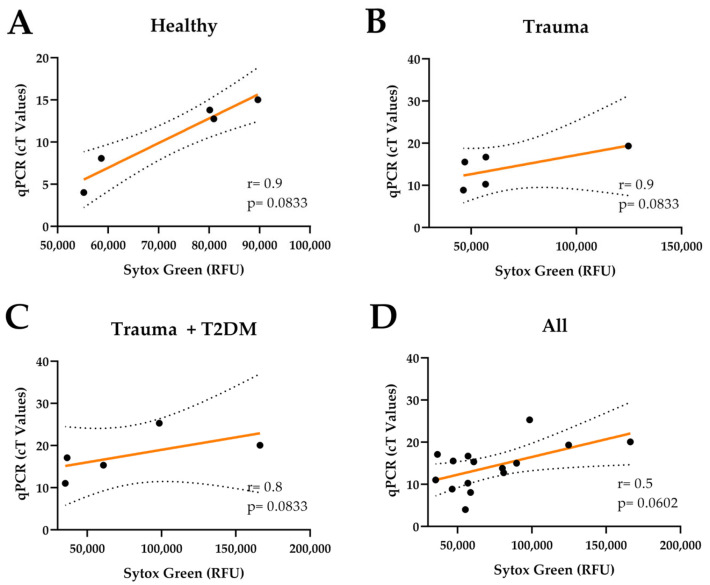
Correlation between the cfDNA quantification using qPCR and fluorometry. The same samples were assessed using the qPCR method and the Sytox Green staining. (**A**) cfDNA from healthy volunteers. N = 5, n = 2; (**B**) cfDNA from trauma patients. N = 5, n = 2; (**C**) cfDNA from trauma patients with T2DM. N = 5, n = 2. (**D**) Correlation of all groups combined. N = 15, n =2. Correlation was performed using Spearman (r). Data points represent the means of the technical replicates. The dotted lines indicate 95% confidence intervals.

After correlating the cfDNA quantification with NET-related proteins, the total cfDNA amount was detected using a fluorometric method to validate the method further. The cfDNA concentrations of the serum samples from each donor were quantified using qPCR and Sytox Green staining. The positive correlation trends were observed, especially when all the samples were analyzed together (*p* = 0.0602) (Figure 6A–D).

## 4. Discussion

Neutrophils are major players in immune response and homeostasis, mediating the host defense against pathogens. In addition to defense mechanisms such as phagocytosis, cytokine, and chemokine mediation, one important mechanism is the release of the NETs [26]. While NETosis is considered a helpful mechanism to eliminate pathogens, it can hinder the process in some situations by adding to chronic inflammation [27]. One of these instances is trauma healing, where neutrophils are already primed because of underlying diseases such as T2DM, therefore resulting in excessive NETosis, which can be detrimental to the host [5]. Therefore, it is important to be able to assess the NET release in patients to evaluate their risk for chronic inflammation and trauma. The clinical research on modulating NETosis grows with time. A clinical study with an ELA2 inhibitor, AZD9668, showed the indirect inhibition of NETs mitigating inflammation [28]. It was also reported in a clinical trial that rituximab and belimumab could indirectly inhibit the NET production in systemic lupus erythematosus [29]. In addition, there are pre-clinical studies that demonstrate possible treatments for excessive NETosis. Methotrexate, a drug that has been used for rheumatoid arthritis for decades, and diphenyleneiodonium chloride, which controls blood glucose, can inhibit NETosis by inhibiting ROS production [30,31]. An emerging approach is using vitamin supplements or antioxidants. It has been shown that flavonoids, vitamin C, or vitamin D can modulate NET release [32,33]. Recombinant human DNase has been studied in the pre-clinical phase as a more direct treatment [34,35,36]. However, it should be kept in mind that abolishing NETosis might result in a dysregulated immune response [36]; thus, clinicians should monitor the at-risk patients closely during treatment.

Here, we propose a method to measure NETosis directly from serum samples. To achieve this, we utilized the quantification of circulating cfDNA by measuring the KRAS gene using qPCR. Measuring circulating cfDNA as a prognosis marker has been a topic of discussion for many years. In patients with septic shock, plasma DNA levels were elevated, and correlated with pro-inflammatory cytokines and organ damage [37]. The quantity of serum and plasma cfDNA in patients with non-small-cell lung cancer reflected the tumor stage and survival [38]. Similarly, a positive association between cfDNA and a bad prognosis was demonstrated in breast cancer [39]. The levels of cfDNA in patients with ovarian cancer were significantly higher than in healthy individuals [40]. Additionally, cfDNA quantification has been suggested as a prognostic and diagnostic approach in neurodegenerative diseases such as multiple sclerosis and amyotrophic lateral sclerosis [41].

Because NETs consist of genomic DNA, in theory, any gene can be used to measure the released NET amount from the serum or plasma. In this study, we selected the KRAS gene as it has been used frequently for circulating cfDNA research [18,42,43].

First, we tested various types of blood samples to determine which would yield the most efficient results. Among serum, EDTA plasma, Li–heparin plasma, and citrate plasma samples, only serum samples provided stable results in the qPCR experiments. Notably, this method does not require any DNA isolation process, making it more convenient. Additionally, serum collection is minimally invasive and can be easily performed during routine blood sampling. Next, we assessed and compared the ELISA and fluorometric methods of NET quantification, which are commonly used. Here, we showed that these methods are not sensitive enough in lower concentrations and are subject to fluctuations in sensitivity with different sample types.

As previously mentioned, T2DM plays a crucial role in the prognosis of chronic inflammation during wound or fracture healing. Therefore, we analyzed trauma patients with inflammation, both with and without T2DM. The absolute quantification of circulating cfDNA revealed that trauma patients with T2DM had significantly higher cfDNA levels in their serum compared with healthy controls and trauma patients without T2DM. It is also noteworthy that individuals with high circulating cfDNA concentrations were observed not only in the T2DM group but also in the healthy and trauma-only groups, potentially indicating a risk of developing complications for these individuals.

Following the quantification of the cfDNA, we measured the protein concentration of NET-related proteins in our patients. For this, neutrophil elastase (ELA2 or NE), myeloperoxidase (MPO), and citrullinated histone 3 (citH3) were selected. ELA2 is an antimicrobial protein that translocates to the nucleus from granules to help cleave histones and initiate chromatin decondensation [44]. MPO is another protein that is required for NETosis and synergizes with ELA2 to modulate nuclear decondensation [24]. During the NET formation, histones are citrullinated to facilitate chromatin decondensation. Therefore, citH3 is another protein that has been used as a NETosis marker [45]. The ELA2 protein levels did not differ between the selected groups. The MPO concentration in the serum was lowest in healthy individuals, whereas it had the highest amount in trauma patients without T2DM. citH3 showed the same trend as circulating cfDNA concentrations, being the lowest in healthy and highest in trauma with the T2DM patient group.

Circulating cfDNA can originate from various biological processes, such as apoptosis or necrosis [30]. To confirm that our method can effectively assess NET release, we analyzed the correlations between neutrophil proteins and cfDNA concentrations. The results revealed a positive correlation between cfDNA levels and all neutrophil-related proteins, with the strongest correlation observed with citH3. It can be discussed that MPO and ELA2 are proteins that can be released independently in NETosis via the neutrophil degranulation mechanism [46], whereas citH3 is more correlated with DNA release. Different neutrophil defense mechanisms can be in play with the patients in this study as this is a heterogeneous cohort that can mimic the clinical settings and demonstrate that the suggested circulating cfDNA quantification method can be useful outside the controlled research environments. citH3 is widely regarded as a reliable biomarker for NETosis and has been frequently utilized in related studies [30,31,32]. Furthermore, citH3 has been shown to be a suitable marker for sepsis-related NET release [33]. These findings support our hypothesis that quantifying circulating cfDNA from serum samples serves as a reliable indicator of NET release.

It is crucial to underline that most of the popular methods used to detect or quantify NET formation are indirect and need another confirmation method, since this way of detection encompasses all cellular DNA. For instance, DNA-intercalating fluorescent dyes are frequently used. However, this method cannot distinguish between NETosis and necrosis. A method where plasma samples are directly stained with Sytox Green was suggested [47], but this method also does not exclude the possibility of DNA release through alternative mechanisms. Additionally, the fluorescence-based quantification of circulating cell-free DNA as a NETosis marker has been employed in predicting the prognosis of patients with severe burn injuries [48] and sepsis [49]. We also utilized this approach with serum samples in this study and showed a correlation trend between the qPCR quantification and the fluorometric analysis. However, the biggest variation from the correlation can be seen in the samples with lower circulating cfDNA concentrations, which can stem from the low sensitivity of the fluorometric quantification. Immunofluorescence microscopy is a good method for visualization, but quantification using microscopy pictures is open to bias and is tedious [50]. Flow cytometry can be a good option for NET quantification, especially in blood samples, although it cannot detect already released NETs and needs sample preparation [20,51]. ELISA is another simple and quantitative option; nonetheless, the markers and standardization are topics of discussion [1,52].

Furthermore, the sample type and the commercial kit might affect the readings, as shown here. A Western blot can be used to specifically check NET-related protein levels, just like ELISA, but the optimization of the protocol and sample quality are essential [1]. Other methods, such as electron microscopy or live imaging, are possible, yet these are sophisticated methods and might not be accessible [1,50,53]. Here, we suggest a robust, quantitative, and accessible method that can be used to detect NET formation from serum samples.

## 5. Limitations

The primary limitation of this method is that it only detects genomic DNA in its current form. It is well established that mitochondrial DNA can also be released during NET release, albeit in much smaller quantities compared with genomic DNA [40]. To address this, another mitochondrial-specific target could be utilized to detect mtDNA more effectively. Even though this method cannot incorporate mitochondrial DNA, it can successfully indicate NETosis levels. Furthermore, the method should be tested on different, more homogeneous cohorts without comorbidities, as our patient groups exhibited high variability in the measurements. To achieve more precise results, serum samples should be processed as soon as possible. Similar to the other NET release detection techniques discussed previously, this method may require validation through an additional complementary method to ensure accuracy and reliability, as circulating cfDNA can be released through different mechanisms.

## Figures and Tables

**Table 1:** Characteristics of the study population. Values are shown as means ± standard deviations.

			Control	Trauma	Trauma + T2DM
Age (Years)			45 ± 15.6	50.7 ± 14.4	62.06 ± 9.2
Gender			14 Female, 13 Male	30 Female, 37 Male	12 Female, 19 Male
Random Blood Glucose (mg/dL)			N/A	100.2 ± 15.4	191.6 ± 63.4
HbA1c (%)			N/A	N/A	7.9 ± 1.1
CRP (mg/L)			N/A	23.7 ± 28.3	49.5 ± 75.6
**Trauma Types**	Bone Complications	Pseudoarthrosis	-	11	11
		Osteomyelitis	-	8	3
		Joint Empyema	-	7	1
		Arthrodesis	-	2	-
		Fracture Infection	-	7	3
	Wound Complications	Wound Infection	-	24	20
		Outer Malleolus Formation	-	1	-
		Foot Necrosis	-	5	3
		Fistula	-	1	-
		Limp Ulceration	-	1	-

**Table 2:** Information on used primers.

Primer	GenBank Accession	Forward Primer (5′–3′)	Reverse Primer (5′–3′)	Amplicon Size (bp)	Ta (°C)
**KRAS**	NC_000012.12	CCTTGGGTTT–CAAGTTATATG	CCCTGACATA–CTCCCAAGGA	67	60

**Table 3:** Information on the ELISA kits.

Target Protein	Order No.	Company	Dilution Factor	Chromogenic Substrate
**α-Defensin**	DY8198-05	R&D Systems	20	Tetramethylbenzidine
**AZU1**	ELH-AZU1	RayBio	5	Tetramethylbenzidine
**citH3**	Cay501620-96	Cayman Chemicals	25	Tetramethylbenzidine
**ELA2**	DY9167-05	R&D Systems	25	Tetramethylbenzidine
**MPO**	DY3174	R&D Systems	25	Tetramethylbenzidine

## Data Availability

Data are available from the corresponding author on request.

## Supplementary Materials

The following supporting information can be downloaded at: https://www.mdpi.com/article/10.3390/mps8020042/s1, Figure S1: Assessment of the age effect on cfDNA concentration and the NETosis-related proteins in the cohort. (A) Correlations of the ages and the cfDNA concentrations of the individuals in this study. Correlation analysis was performed using pairwise Spearman correlation (r). Healthy N = 27, Trauma N = 67, Trauma + T2DM N = 31. (B) Age distribution of age-matched participants. (C) The concentration of cfDNA in serum samples was determined using qPCR for the KRAS gene. Healthy N = 15, n = 3; Trauma N = 15, n = 3; Trauma + T2DM N = 15, n = 3. (D) Protein concentrations were measured from serum samples with respective ELISA kits. MPO: Healthy N = 6, n = 2; Trauma N = 15, n = 2; Trauma + T2DM N = 15, n = 2. ELA2: Healthy N = 6, n = 2; Trauma N = 15, n = 2; Trauma + T2DM N = 15, n = 2. citH3: Healthy N = 4, n = 2; Trauma N = 4, n = 2; Trauma + T2DM N = 6, n = 2. Statistical analysis was performed using the Kruskal–Wallis test. * *p* < 0.05 as indicated. Figure S2: Correlation of the protein concentrations quantified using ELISA and cfDNA concentrations quantified using qPCR. Correlation coefficients were calculated using the Spearman method in R for complete observations. (A) Healthy participants, N = 8. (B) Trauma patients, N = 15. (C) Trauma patients with T2DM, N = 15.
