# Supplementary material for: Quantification of Circulating Cell-Free DNA as a NETosis Marker in Trauma Patients with Type 2 Diabetes Mellitus
Source: Methods Protoc. 2025 Apr 16;8(2):42. doi: 10.3390/mps8020042 (PMC12029683; doi:10.3390/mps8020042)
Supplement: Supplementary file 1 [file mps-08-00042-s001.zip › mps-3481158-supplementary.pdf]

Supplementary Figure S1:

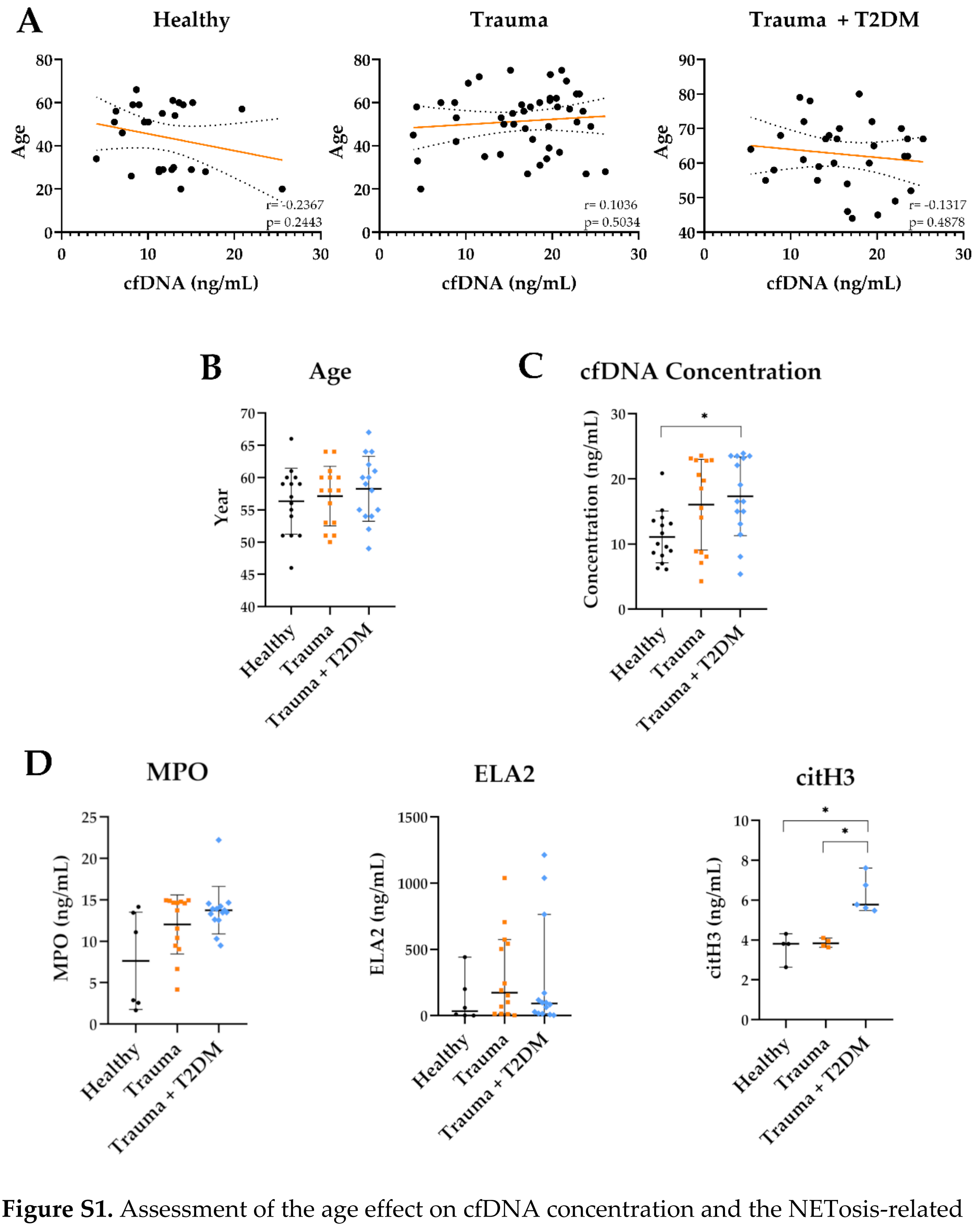

Figure S1. Assessment of the age effect on cfDNA concentration and the NETosis-related proteins in the cohort. (A) Correlations of the ages and the cfDNA concentrations of the individuals in this study. Correlation analysis was done by Pairwise Spearman correlation (r). Healthy N=27, Trauma N=67, Trauma + T2DM N=31. (B) Age distribution of age-matched participants. (C) The concentration of cfDNA in serum samples was determined by qPCR for the KRAS gene. Healthy N=15, n=3, Trauma N=15, n=3, Trauma + T2DM N=15, n=3. (D) Protein concentrations were measured from serum samples with respective ELISA kits. MPO: Healthy N=6 n=2, Trauma N=15, n=2, Trauma + T2DM N=15 n=2. ELA2: Healthy N=6 n=2, Trauma N=15, n=2, Trauma + T2DM N=15, n=2. citH3: Healthy N=4 n=2, Trauma N=4

n=2, Trauma +T2DM N=6, n=2. Statistical analysis was done by the Kruskal-Wallis test. * $p < 0.05$ as indicated.

Supplementary Figure S2:

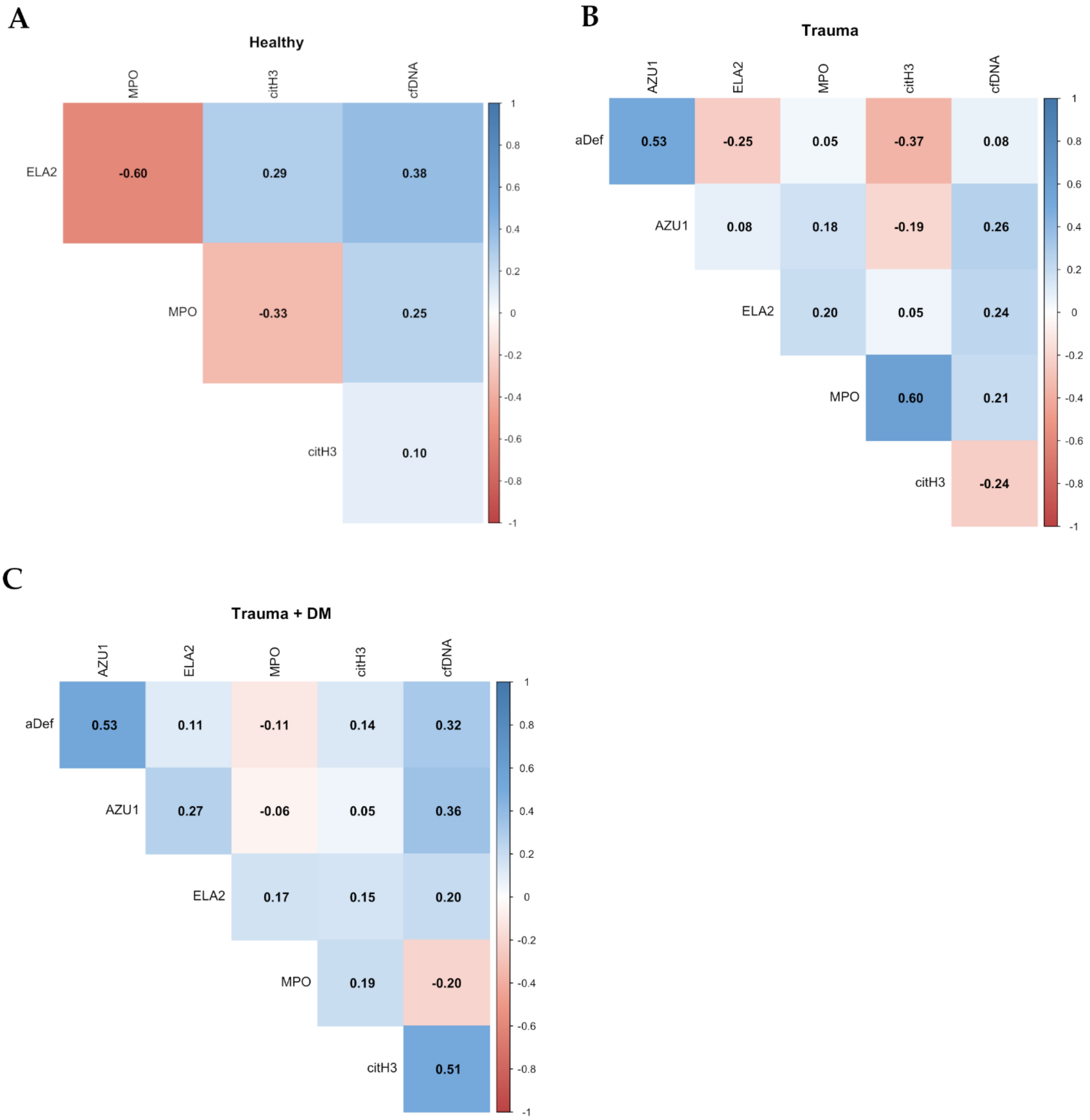

Figure S2. Correlation of the protein concentrations quantified by ELISA and cfDNA concentrations quantified by qPCR. Correlation coefficients were calculated with the Spearman method in R for complete observations. (A) Healthy participants, N=8. (B) Trauma patients, N=15. (C) Trauma patients with T2DM, N=15
